# Active inference and speech motor control

**DOI:** 10.3758/s13423-026-02871-1

**Published:** 2026-04-20

**Authors:** Abigail R. Bradshaw, Clare Press, Matthew H. Davis

**Affiliations:** 1 MRC Cognition and Brain Sciences Unit, University of Cambridge, 15 Chaucer Road, Cambridge, CB2 7EF UK; 2 Department of Experimental Psychology, University College London, 26 Bedford Way, London, WC1H 0AP UK; 3 Department of Imaging Neuroscience, University College London, 12 Queen Square, London, WC1N 3AR UK

**Keywords:** Speech motor control, Active inference, Predictive coding, Sensorimotor interactions, Sensorimotor learning

## Abstract

Active inference is a domain-general theory of brain functioning which reconceptualises the perception–action interface in terms of a common process of minimization of sensory prediction errors. Such accounts have been extensively applied to the control of manual action guided by visual sensory feedback; however, they have received relatively little explicit attention in speech motor control. This is despite speech providing a critical test case, arguably being one of the most crucial and intricate of human sensorimotor functions. The application of active inference to speech motor control can allow crosspollination of decades of work from neighbouring disciplines, and could highlight where speech motor control mechanisms may be similar to, or differ from, those in other motor control domains, by establishing mechanistic explanation in common terms. We present here the first detailed description of an active inference framework of auditorily guided speech production. We compare the architecture of active inference models to existing computational models of speech motor control, and describe an active inference account of how compensation and adaptation result from perturbations of auditory feedback. We highlight several unique aspects of active inference, as well as emerging hypotheses for future empirical work. In particular, active inference accounts emphasise a role for proprioception in speech motor learning, and offer the potential to model the effects of other voices on speech production in phenomena such as phonetic convergence.

Perception and action during speech are intimately interwoven; to state the obvious, a speech action is always accompanied by perception—of the sound of our own voice and the somatosensations elicited by movements of our lips and tongue. Speech is thus an inherently sensorimotor act, relying on tight couplings between sensory and motor representations. As such, models of speech motor control place great emphasis on the use of auditory and somatosensory feedback. A particular focus is placed on prediction of expected sensory signals during speech production, with prediction errors being used to inform and modify movements (Guenther 2016; Guenther et al., 2006; Houde & Nagarajan, 2011; Parrell & Houde, 2019; Tourville & Guenther, 2011).

In a somewhat separate literature, a reconceptualisation of the perception–action interface has been proposed in ‘predictive coding’ and ‘active inference’ frameworks (Adams, Shipp, et al., 2013a, 2013b; Clark, 2013; Friston, 2010). These domain-general neurally grounded frameworks fuse the mechanisms underlying perception and action into a single computation; the minimisation of prediction errors. Predictive coding conceives of perception as a form of inference, in which the brain must reconstruct an internal model of the outside world (a generative model) from noisy sensory input. This relies on a neural architecture in which descending/top-down pathways convey predictions, and ascending/bottom-up pathways convey prediction errors (i.e., the discrepancy between predicted and received sensory input; Rao & Ballard, 1999). Such prediction errors are used to update the generative model, such that subsequent sensory predictions (or, in the context of a generative model, beliefs about states in the outside world) become more accurate, thereby minimising future prediction error. This is known as perceptual inference. For example, if we predict that the shape approaching us in the park is a dog, but then receive sensory evidence that mismatches this prediction (e.g., visual features that indicate it is in fact a fox), this prediction error is used to update our generative model (i.e., so that we correctly perceive the moving shape as a fox), and this will obligatorily change predictions for other perceptual experiences (e.g., foxes make screaming as well as barking sounds) and other events (since we might expect to encounter foxes in this park in future).

Crucially, these prediction error minimisation accounts have been extended to suggest that action also arises from the same computation; whereas perceptual inference involves adjusting predictions to fit sensory evidence, so called ‘active inference’ involves acting on the environment to change sensory evidence to conform with predictions (see Fig. 1). For example, when grasping a cup, the brain predicts the sensory (e.g., tactile, visual, proprioceptive) input that would arise if that action was performed; the resulting prediction errors then drive action (i.e., contractions of muscles) to fulfil these predictions. In this way, both perception and action are reduced to a common process of prediction error minimisation, operating on a shared set of predictions (i.e., the same generative model). So far, these accounts have been extensively discussed and researched in relation to the domains of manual action and visual sensory feedback, and continue to be debated (Friston 2011; Friston et al., 2011; Limanowski & Friston, 2020; Yon et al., 2018). Within these domains, active inference has been applied to a diverse range of processes, including body ownership (Limanowski, 2022b), learning (Friston et al., 2016), attention (Brown et al., 2011), and illusions (Brown et al., 2013), as well as different psychopathologies (Paulus et al., 2019) such as schizophrenia (Adams, Stephan, et al., 2013) and Parkinson’s disease (Friston et al., 2012).

**Fig. 1:**
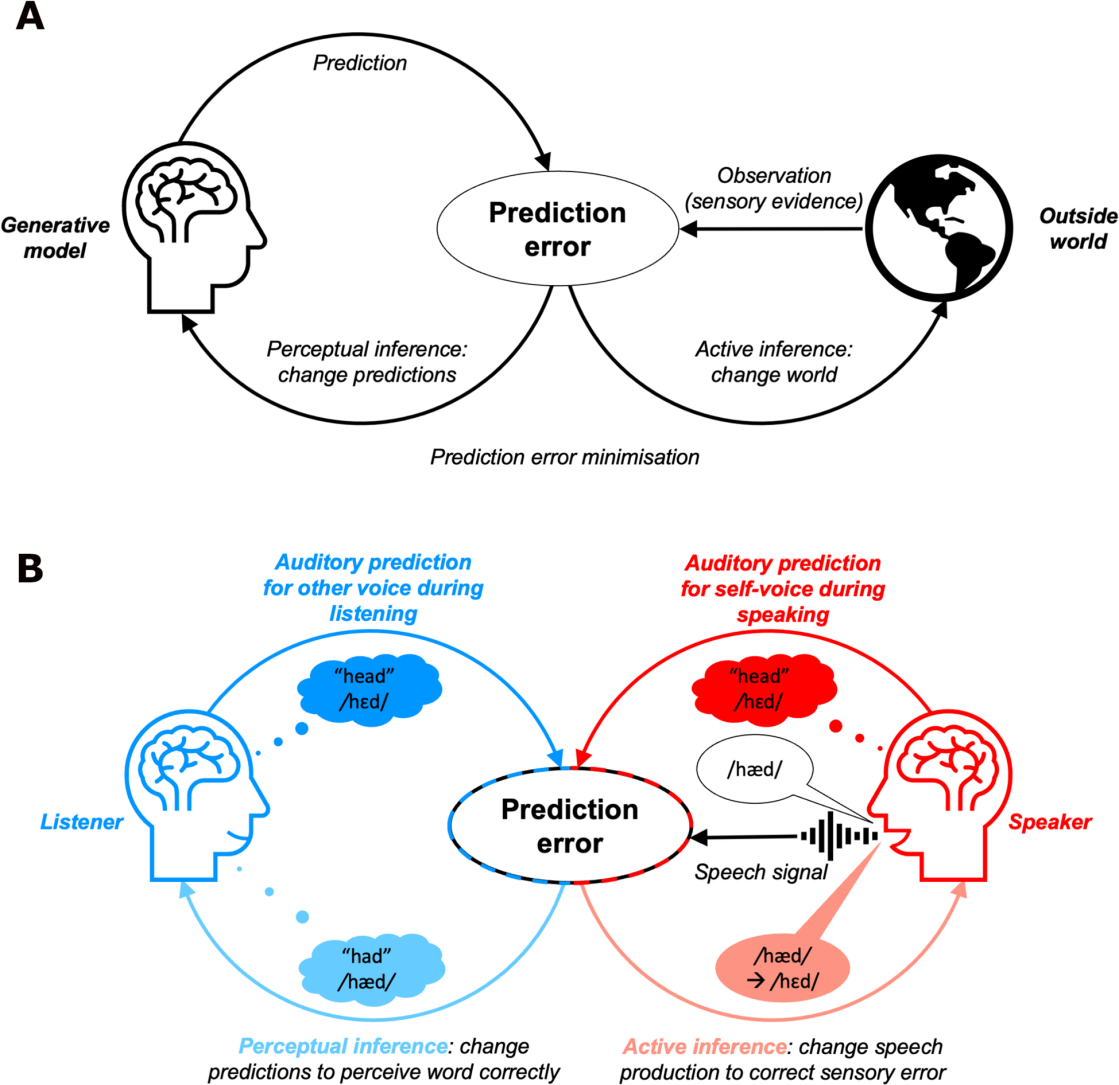
Schematic of perceptual inference versus active inference. (**A**) Active inference accounts unify the processes of perception and action into a common process of prediction error minimisation. During perception, we minimise prediction error by changing our generative model to better predict the sensory evidence that we obtain from the outside world. During action, we ensure that sensory input conforms to what we predict by acting to achieve our desired state of the world. Adapted from Parr et al. (2022). **B** Application of this perceptual inference and active inference framework to speech perception and production. A listener (left) predicts the speech signal /hɛd/ associated with the word “head” and compares this prediction (dark-blue arrow) to the incoming speech signal (black arrow). Any resulting prediction error (e.g., hearing speech with a different vowel /hæd/) can be minimised by perceptual inference (light-blue arrow) either by determining that the word “had” was spoken instead or updating the listener’s predictions for the sound of the critical vowel /ɛ/ (i.e., perceptual learning). A speaker (right) performs active inference; they move their vocal tract to generate speech that satisfies their predictions for the sound of the word “head” (minimising prediction error through action). If comparison of the speaker’s prediction (red arrow) with auditory feedback from their own voice (black arrow) elicits a prediction error (e.g., they hear /hæd/ instead due to incorrect positioning of the vocal tract or due to an external auditory perturbation), this is minimised through active inference (pink arrow) by changing speech production so that the correct vowel is produced. (Colour figure online)

Within the domain of speech, despite applications of predictive coding frameworks to understanding speech perception (Ar nal & Giraud, 2012; Friston et al., 2021; Sohoglu & Davis 2020), active inference accounts have received relatively little (explicit) attention in speech motor control. It is thus unclear whether predictive coding accounts are compatible with existing models of speech motor control, and whether behavioural evidence from the study of speech production might support or contradict assumptions of active inference accounts. Given these accounts claim to present principles that apply to perception and action across all domains, their application to speech is likely to both facilitate the field’s understanding of speech production and perception, as well as providing a critical test case for active inference accounts more broadly. The development of active inference accounts for speech motor control is an appealing prospect, given their potential to offer a unifying framework within which to consider a wide range of phenomena in speech, from sensorimotor learning, to perceptual learning, sense of agency over a voice, social aspects of vocal communication and disorders of speech; as well as the potential to better integrate processes of perception and action in speech with those in other domains. As a starting point, Fig. 1B presents a schematic illustration of the application of active inference to speech in the context of an interaction between a speaker and a listener; here, speech perception and production are unified into a shared computational process of prediction error minimisation.

This review therefore seeks to bridge the gap between existing theories and models in speech motor control and predictive coding accounts of action and perception. At the surface level, both fields use similar terminology; however, these superficial similarities might mask more critical theoretical differences leading to different predictions for empirical work. Before we begin our review, we give a glossary of terms that are commonly used in speech motor control and predictive processing theories. The subsequent review section first compares the architecture of these models, to explain how active inference accounts differ from standard approaches in speech motor control. Second, we consider whether predictive coding accounts can accommodate key behavioural phenomena in the sensorimotor control of speech, with a particular focus on auditorily guided speech motor control. Specifically, we present an active inference account of compensation and speech motor adaptation to auditory feedback perturbations. Finally, we address outstanding areas requiring further research to support the refinement and assessment of predictive coding and active inference accounts of speech motor control. 

## Glossary of terms in speech motor control and predictive processing:

- **Feedback control:** A mode of motor control in which motor commands are generated ‘online’ through a comparison of predictions with sensory feedback.
- **Feedforward control:** A mode of motor control in which motor commands are preplanned and executed without reference to sensory feedback.
- **Prediction error:** The discrepancy between a predicted and an observed sensory signal.
- **Perceptual inference:** A process for minimising prediction errors by updating predictions to match sensory feedback.
- **Active inference:** A process for minimising prediction errors by acting on the environment to bring the sensory world in line with predictions.
- **Proprioception:** Sensory information that signals the current state of muscles and joints, both at rest and during movement.
- **Efference copy and corollary discharge:** Mechanisms for generating predictions of the expected sensory consequences of movements. Although historically used somewhat interchangeably, efference copy generally implies a particular mechanism in which sensory predictions are obtained via a ‘copy’ of the motor commands sent out by motor cortex; a forward model (that specifies mappings between motor commands and sensory outcomes) is then used to obtain the predicted sensory consequences of those commands. These can be sent to sensory areas to allow computation of prediction errors through comparison with incoming sensory input. Active inference renders such efference copies per se unnecessary, since motor commands are themselves sensory (proprioceptive) predictions (and thus there is no need for transformation from motor to sensory reference frames); instead, sensory predictions in other domains are described as corollary discharges.
- **Generative model:** An internal model that encodes beliefs about the state of the outside world; specifically, the probability of observing sensory inputs given certain hypothesised ‘causes’. This is used to generate predictions about expected sensory inputs that can then be compared to observed sensory feedback to compute prediction errors. These prediction errors are then used to update the model and infer the most likely cause of current sensations, from among many possible causes. For speech motor control, a generative model will encode beliefs about mappings from proprioceptive (causes) to auditory sensations.
- **Model inversion:** Inversion of the generative model refers to inferring a proprioceptive sensory target from an auditory target, using mappings between these two sensory spaces (between proprioceptive states or ‘causes’ and their associated auditory outcomes). Conventional motor control theories require the use of inverse models to implement the complex transformation from a desired movement trajectory or sensory outcome (in extrinsic coordinates) to motor commands (in intrinsic coordinates). This is a known challenge in engineering and computational modelling of motor control (Friston, 2011; Wolpert et al., 2011). Conversely, in active inference, the generative model can generate both exteroceptive (e.g., auditory) and interoceptive sensory (i.e., proprioceptive) predictions for a desired movement (e.g., associated with a desired speech sound); the relevant inverse mapping that drives action is thus simply that between proprioceptive predicted sensations and muscle movements, which can be straightforwardly implemented by reflex arcs at the periphery (Adams, Shipp, et al., 2013a, 2013b). In this way, active inference claims to solve the hard inverse problem in motor control (Friston, 2011). The problem of acoustic-to-articulatory inversion and how it can be modelled has posed a significant challenge and is an ongoing area of research (Anumanchipalli et al., 2019; Kello & Plaut, 2004; McGhee et al., 2024; Uria et al., 2012; Wang et al., 2022); however, mappings between proprioceptive causal states and auditory outcomes are also complex, and how the generative model learns and infers these mappings in the context of speech will be an important challenge for active inference accounts of speech motor control to solve (Najnin & Banerjee, 2017).
- **Sensory perturbation paradigm:** An experimental paradigm in which sensory feedback in a particular modality (e.g., audition) is replaced during action (e.g., speaking) with an altered version. By using near-real-time signal processing, the apparent sensory consequences of a movement appear altered or ‘perturbed’.
- **Compensation:** Reactive changes made to an ongoing action to correct for an unexpected or random perturbation of sensory feedback.
- **Adaptation:** A form of sensorimotor learning, in which the motor control system learns a new mapping between movements and sensory outcomes (e.g., between movements of the speech articulators and auditory or somatosensory feedback). Unlike compensation, adaptation is gradually acquired in response to sustained perturbations of sensory feedback and can persist after sensory perturbation is removed.

## Comparing active inference theories to existing models of speech motor control

**Fig. 2:**
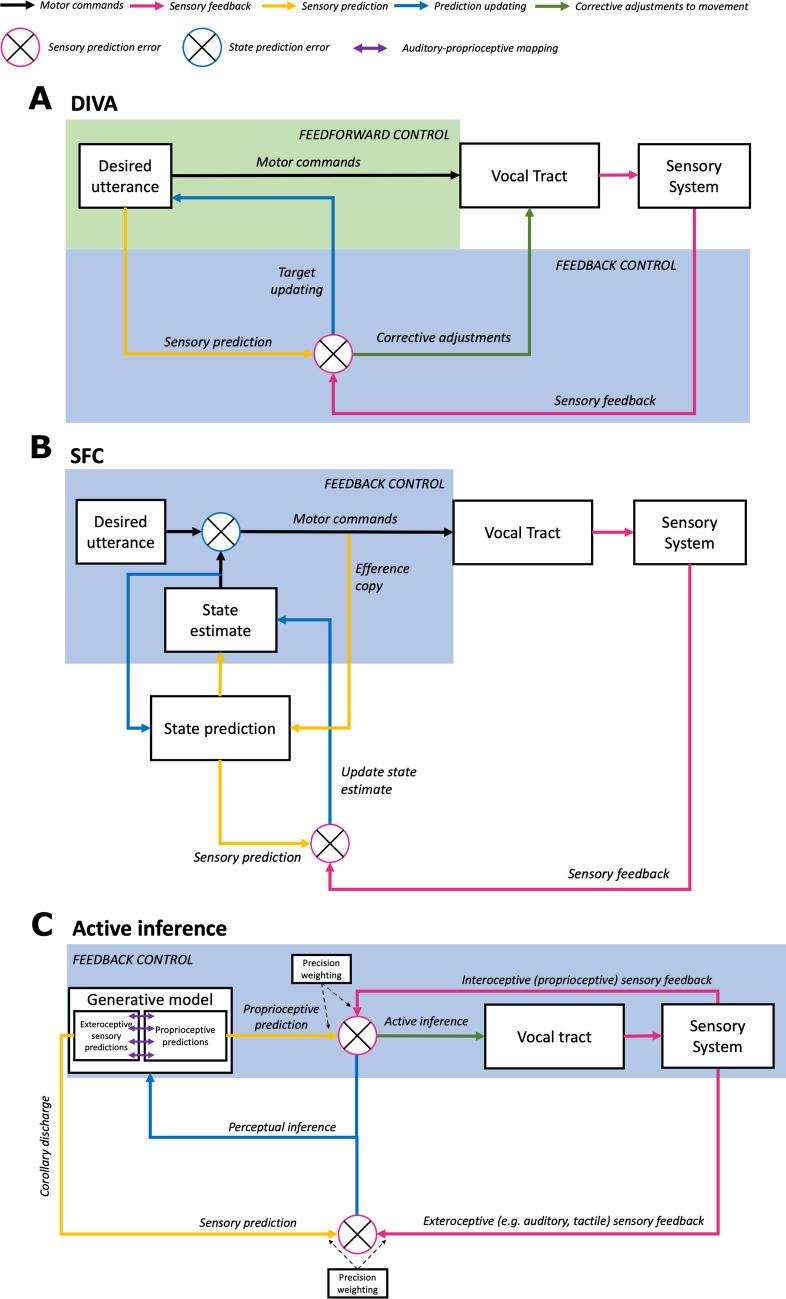
Models of speech motor control. Comparison of (**A**) the DIVA model, (**B**) state feedback control (SFC), and (**C**) active inference framed in the context of speech motor control. These schematics have been created with the aim of facilitating comparison between the models, and thus various simplifications or translations of terminology have been performed. It should be noted that both DIVA and SFC models have separate control systems for auditory and somatosensory feedback; for purposes of clarity, these have been combined in these schematics, since the underlying operations are the same. Conversely, active inference makes a distinction between mechanisms for proprioceptive (interoceptive) sensory feedback and all other types of exteroceptive sensory feedback (e.g., auditory, tactile), and so these systems are shown separately in panel **C**. Circles with *X*s inside denote ‘comparators’ which compute prediction errors. (Colour figure online)

Figure 2A and B illustrate the architecture of two dominant, but distinct, models of speech motor control; the directions into velocities of articulators (DIVA) model (Guenther 2016; Guenther et al., 2006; Tourville & Guenther, 2011); and the state feedback control (SFC) model (Houde & Nagarajan, 2011). We chose to restrict our comparison with active inference to these two models, since these have been the most influential in the literature over the last few decades (for a full review of current models of speech motor control, see Parrell, Lammert, et al., 2019a, 2019b). Figure 2C illustrates the computational architecture of predictive coding/active inference theories as applied to speech motor control. A summary of key differences between these models is given in Table 1.

**Table 1:** Comparison of traditional models of speech motor control with the active inference framework

Model	Feedback (blue box) or feedforward (green box) motor control	Mechanism for generating motor commands (black arrows)	Source of sensory predictions to be compared with sensory feedback (yellow arrows)	Mechanism(s) for minimising prediction errors (PE) (blue and green arrows)
*DIVA* **(**Guenther, 2016; Guenther et al., 2006**)**	Both	Generated by summing the output (motor commands) generated by the feedforward and feedback controllers.	Activation of a desired utterance generates sensory targets (predictions), which are directly compared with sensory feedback.	Single PE between desired and observed sensory feedback can be minimised via two routes: (1) Minimised directly through action (feedback control, green arrow) and (2) minimised by updating feedforward predictions if prediction errors are consistent (blue arrow).
*SFC* Houde and Nagarajan, 2011	Feedback only	Generated by comparing a desired ‘state’ of the articulators (e.g., their positions and velocities) to an estimate of the current state.	Efference copy of motor commands generates a state prediction (belief about the state of the articulators) which can be used to derive sensory predictions for comparison with sensory feedback.	Two types of PEs are minimised. PE between sensory prediction and sensory consequences is minimised through updating the predictions (blue arrows). PE between desired state and state estimate is then used to generate motor commands (to minimise PE, black arrow).
*Predictive coding and active inference* **(Current paper)**	Feedback only	Motor commands are replaced by proprioceptive sensory predictions fulfilled through motor reflexes.	Generative model (beliefs about the state of the world) generates sensory predictions for comparison with sensory feedback.	Two routes for minimisation: (1) Minimised through action that forces sensory feedback to conform to predictions, ultimately achieved by updating proprioceptive predictions which are fulfilled by reflex arcs (active inference, green arrow) and (2) minimised by updating predictions in the generative model (perceptual inference, blue arrows).

### Different mechanisms for movement generation

A key architectural difference between these three models concerns the mechanisms underlying movement generation. The DIVA model (Guenther 2016; Guenther et al., 2006; Tourville & Guenther, 2011) proposes that speech movements are generated from the combined output of a feedback and feedforward control system. In feedback control, motor commands are generated ‘online’ through a comparison between predicted and actual sensory feedback. This process can allow action planning to be sensitive to errors in sensory feedback that indicate the movement did not achieve the expected sensory outcome. However, on its own, such a feedback control system would be highly restricted in the speed of movements it can generate, due to delays in the availability of sensory feedback. DIVA solves this problem by combining feedback control with feedforward control. In a feedforward control system, speech motor commands are fully pre-planned and unfold in a pre-specified manner, without regard to sensory feedback. By using a combined feedback and feedforward architecture, this thus allows DIVA to employ both feedforward control to support rapid articulation of connected speech, and feedback control to enable sensitivity to errors in sensory feedback. According to DIVA, the feedback controller can ‘teach’ the feedforward controller, using sensory prediction errors to update sensorimotor mappings ‘offline’ (see blue arrow in Fig. 2A). This process facilitates a developmental shift from an early reliance on feedback control (before stable sensorimotor mappings have been learnt) to an almost exclusive reliance on feedforward control in the mature system.

SFC (Houde & Nagarajan, 2011) provides an alternative solution to the problem of slow sensory feedback within an architecture that always invokes feedback control. Here, motor commands are generated ‘online’ through the comparison of an *estimate* of the current state of the articulators (e.g., positions and velocities of the lips, tongue, jaw) with a desired state. Crucially, such a state estimate is informed not only by sensory feedback, but also by a prediction of expected sensory feedback based on an ‘efference copy’ of the motor commands. This prediction provides a faster means of estimating the current state of the articulators than reafferent sensory feedback, enabling new motor commands to be generated before external sensory feedback is available. In this way, while SFC relies on state estimation to compute error ‘internally’ (the difference between a desired and *estimated* state of the articulators), feedback control in DIVA relies on ‘external’ errors (the difference between desired and current sensory feedback); there is thus no need for explicit state estimates in DIVA, as the relevant comparison with sensory predictions is made directly with sensory feedback. This means that, in contrast to DIVA, within the SFC framework sensory feedback can only indirectly affect the generation of motor commands, through updating of the state estimate (i.e., there are no green arrows by which sensory prediction errors can directly inform movement in Fig. 2B). In both the DIVA and SFC models, however, sensory predictions are distinct from the motor commands themselves.

Conversely, active inference radically reimagines the concept of motor commands by equating them with sensory predictions. This builds on the ideomotor theory of action originally proposed by William James (1890), who argued that all movements are represented in terms of their effects. In a similar vein, active inference proposes that the intention to act begins with the brain predicting the sensory outcomes associated with performance of that action (e.g., visual, auditory, somatosensory feedback); action is then employed to minimise the resulting prediction errors and bring about the predicted sensory feedback. Generation of the movement itself, however, relies not on motor commands, but on proprioceptive predictions of the internal sensations signalling the position or state of the muscles and joints across a movement trajectory, such as the stretching of muscle spindles (yellow arrow in Fig. 2C). Such predictions are generated in primary motor cortex (through interactions with other cortical and subcortical areas as in conventional motor schemes, such as premotor cortex, the basal ganglia and cerebellum) and then conveyed via descending projections from (Betz) pyramidal cells to motor neurons at the periphery (e.g., within the spinal cord; Parr et al., 2021, 2025). Movements of the muscles are generated via comparison of these predictions with actual proprioceptive feedback at the periphery. Specifically, classical reflex arcs are used to drive activity in motor neurons to minimise these proprioceptive prediction errors (i.e., to generate movement that brings about proprioceptive sensations that match predictions; Adams, Shipp, et al., 2013a, 2013b; Friston, 2011; Limanowski & Friston, 2020).

**Fig. 3:**
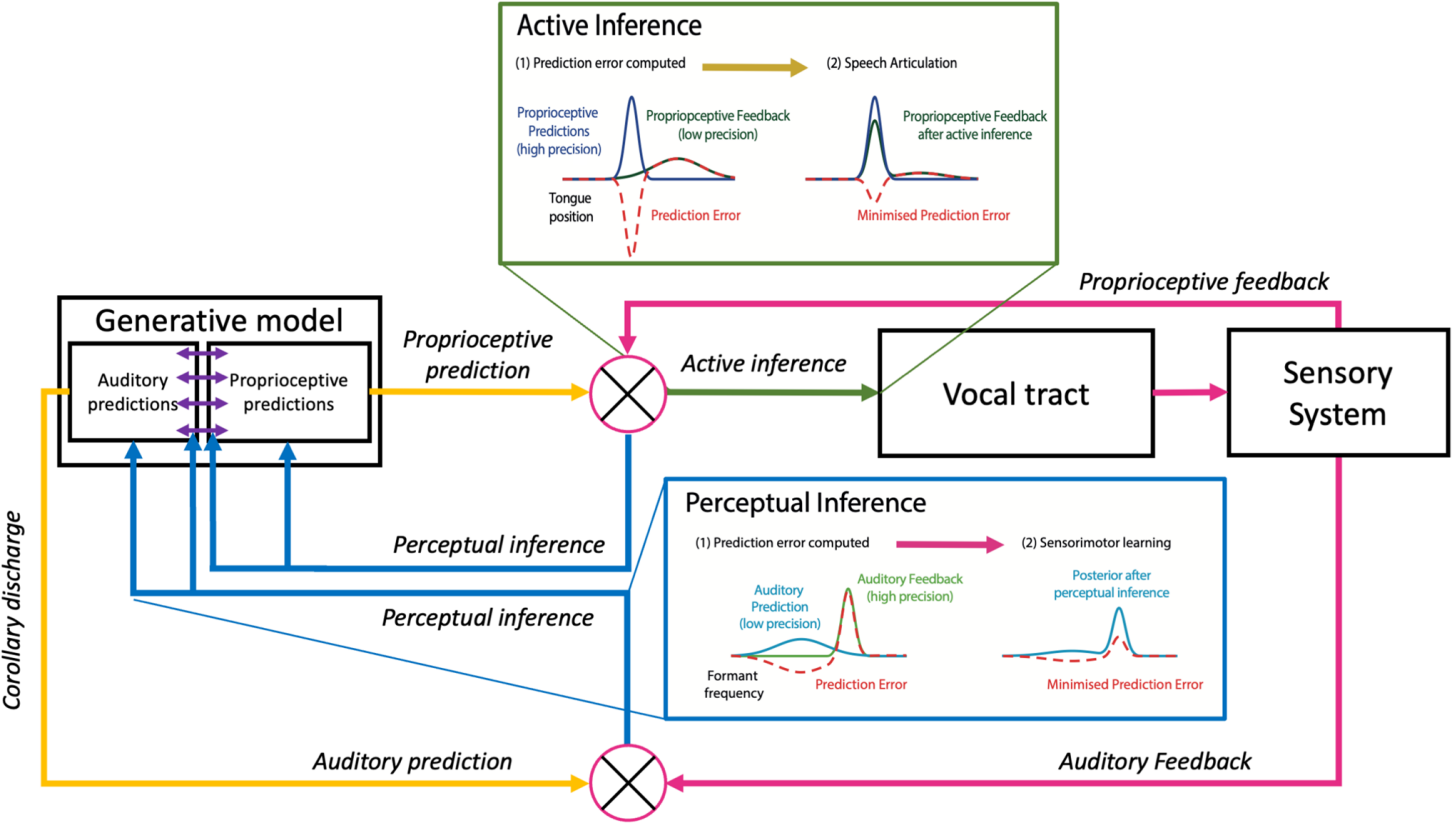
An illustration of the use of active inference for proprioception and perceptual inference for audition. Green box illustrates the minimisation of proprioceptive prediction error through active inference, by driving changes to muscle spindles so as to generate proprioceptive feedback to match that predicted. Here, the proprioceptive prediction after the inference process (known as the posterior) remains unchanged. Blue box illustrates the minimisation of auditory prediction error through perceptual inference, whereby the auditory prediction is updated to match the feedback (i.e., generating a posterior representation after inference that has moved closer to the feedback). Such a process would also apply to other forms of exteroceptive sensory feedback (e.g., tactile, visual). Note that prediction errors in sensory domains other than proprioception can also be minimised via action, by engaging proprioceptive predictions in motor cortex (linked to auditory predictions through the generative model, purple arrows), to generate proprioceptive prediction errors at the periphery that are suppressed via active inference. (Colour figure online)

In this way, sensory prediction errors in exteroceptive modalities at the cortical level (e.g., vision, audition) are ultimately unpacked into proprioceptive prediction errors at the level of the peripheral nervous system, which engages lower-level reflexes to drive movement (Shipp et al., 2013). The process of minimising auditory prediction errors via active inference therefore relies on a multimodal integration with proprioceptive predictions to drive proprioceptive prediction errors that can generate movement via reflex arcs. In this way, auditory prediction errors have a more indirect effect on action than in the DIVA model (note the lack of green arrows from auditory prediction errors in Fig. 2C). The use of active inference to generate movement by minimising proprioceptive prediction errors is illustrated in Fig. 3, along with an example of the use of perceptual inference for minimising auditory prediction errors.

Overall, therefore, all descending projections within the brain and down to the peripheral nervous system (see yellow arrows in Figs. 2C and 3)—be they from ‘motor’ or ‘sensory’ areas—are proposed to predict different forms of sensory feedback based on the generative model. Active inference refers to these projections as ‘corollary discharge’ (see Glossary), arguing against the need for an efference copy mechanism per se; that is, sensory (e.g., auditory) predictions do not rely on a ‘copy’ of the motor commands, since the motor commands themselves are sensory (proprioceptive) predictions. It has been argued that such a conceptualisation of descending projections from motor cortex provides a better explanation of its functional anatomy (Adams, Shipp, et al., 2013a, 2013b; Shipp et al., 2013). First, active inference can explain the relative lack of granular cells in layer IV of primary motor cortex, whose role in sensory cortices is to receive sensory reafference; this is attributed to the fact that proprioceptive prediction errors are resolved through action at the periphery. Thus, the forward pathway through which they would ascend back to motor cortex for minimisation via perceptual inference is rendered redundant (although we also note that proprioceptive prediction errors can engage perceptual inference through interactions with primary somatosensory cortex). Second, the anatomical and physiological properties of descending motor projections are more like those of backward connections in sensory cortices (that convey predictions) than forward connections (Adams, Shipp et al., 2013a, 2013b; Shipp, 2005). This is at odds with a more traditional conceptualisation of such projections as conveying instructive motor commands, which would require forward-type properties (e.g., providing driving, linear, and context-independent inputs that predominantly originate in supragranular layers and terminate in Layer 4; for a review of such properties, see Adams, Shipp, et al., 2013).

There is thus a conceptual parallel between the SFC and active inference models, in that movement is generated ‘online’ through a comparison of a desired articulatory state and an estimate of the current state. In this sense, both SFC and active inference employ a ‘feedback control’ strategy for motor control. However, while SFC avoids the issue of delayed sensory feedback by allowing efference copy to inform the state estimate, active inference assumes the estimate is directly based on proprioceptive feedback. Interestingly, proprioception for speech has been shown to operate at faster latencies than auditory speech feedback (Gomi et al., 2002; Nakahara et al., 2004; Sakamoto et al., 2010). For example, using electromyography, Gomi et al. (2002) reported muscle and effector response latencies as rapid as 20 ms to an articulatory perturbation of the jaw, shorter than the latencies typically reported for responses to auditory perturbations of around 100–150 ms (Bauer et al., 2006; Burnett et al., 1997). The issue of sensory feedback delays has been previously addressed in an active inference account of oculomotor control, in which sensory delays can be internally simulated and corrected by the system (Perrinet et al., 2014). Relatedly, active inference accounts also highlight and solve the issue of motor delays in descending projections from motor cortex, through rapid classical reflex arcs that drive movement by minimising proprioceptive prediction errors. For limb motor control these are implemented by alpha motor neurons in the ventral horn of the spinal cord; for oculomotor control (and presumably vocal control) these can be implemented at the level of cranial nerve nuclei. The proponents of active inference argue that such reflex arcs provide a more biologically plausible mechanism by which sensory (proprioceptive) outcomes can be mapped to action (Friston, 2011; Najnin & Banerjee, 2017).

The involvement of proprioception in speech motor control has long been acknowledged (Guenther et al., 1998), although its precise role and importance remains debated. For example, in the DIVA framework, Guenther (2016) argues that learning of auditory targets is the primary driver of speech motor development, with learning of somatosensory (including proprioceptive) targets coming later. Other models place proprioceptive feedback as more central, such as the GEPPETO model in which speech movements are not possible without proprioceptive feedback on muscle length (Perrier et al., 2006). A recent review by Kent (2024) highlights the importance of somatosensation for speech production, contributed to by proprioceptive, tactile, and baroreceptive sensations. Together, these sources of sensory input inform an estimate of the status of the multiple subsystems serving speech (respiratory, laryngeal, and supralargyngeal), both at rest and during movement (Haggard & de Boer, 2014). While there is some debate over which speech articulators contain muscle spindles innervated by the trigeminal nerve (e.g., lips, tongue, pharynx, larynx), most appear equipped with their own special types of receptors which convey proprioceptive information via the facial nerve (Cobo, 2017; Kent, 2024).

A number of studies have attempted to investigate the effect of short-term deprivation of somatosensation on speech production (e.g., by inducing short-term anaesthesia in the articulators or trigeminal nerve block; Casserly & Marino, 2024; De Letter et al., 2020; Niemi et al., 2006; Putman & Ringel, 1976; C. M. Scott & Ringel, 1971). In his review, Kent (2024) summarises this research as finding high individual variability in effects on speech production (e.g., on intelligibility), from negligible to significant. He also notes some limitations of this body of work, including small sample sizes, the relative lack of data from children, and a potential failure rate of 20–25% for inferior alveolar nerve block (located on the mandibular branch of the trigeminal nerve; Khalil, 2014). As noted by Parrell and Houde (2019), it is also probable that the manipulations used in many of these studies mainly affect tactile sensation, with proprioception likely remaining intact. It is therefore difficult to test the necessity of proprioception for speech motor control, making this claim of active inference difficult to evaluate at this time.

### Sensory predictions and prediction error computations

All three models include ‘sensory prediction errors’—that is, the error between a predicted and an observed sensory signal (see circles in Fig. 2 and red prediction error distributions in Fig. 3). The source of these sensory predictions, and the psychological constructs onto which they map, however, differs between these theories. A key distinction between DIVA and SFC concerns whether sensory predictions are derived from *desired* sensory outcomes (as in DIVA), or *beliefs* about the state of the speech system (as in SFC). In DIVA, the sensory prediction is based solely on a stored sensory target associated with the desired utterance that has been formed through learned experience. Such a target does not consist of a single point in time, but of a ‘time-varying region’. Only sensory feedback that falls outside of this region will be detected as a prediction error and thus corrected; conversely, any feedback that falls within the boundaries of this target region will yield equivalent (null) prediction errors. Conversely, in SFC, sensory predictions are derived from the current state prediction, which in turn is based on an efference copy of the motor commands sent to the articulators. This means that sensory predictions are more precise and relate to the specific motor command generated at that moment. Thus, any deviation from this prediction will generate a prediction error, regardless of its relation to categorical boundaries between speech sounds. Comparison with a desired state is instead implemented in SFC as a distinct ‘state prediction error’ (see blue PE circle in Fig. 2B), which compares an estimate of the current state of the articulators (based on efference copy) to this desired state. Crucially it is this state prediction error, and not the sensory prediction error, that is used to compute motor commands and drive movement.

Active inference accounts have provoked much debate concerning their radical reduction of the psychological distinction between beliefs and desires to the single concept of prediction (Smith, Ramstead & Kiefer, 2022; Yon et al., 2020). That is, both beliefs and desires are subsumed into a single set of predictions that make up the generative model (an organism’s internal model of how sensory signals are generated by causes in the outside world). Note how this is reflected in Fig. 2C, which lacks a separate box labelled ‘Desired utterance’ (present in Figs. 2A and 2B for DIVA and SFC). Instead, desired sensory outcomes are ‘simply those that an agent believes, a priori, it will obtain’ (FitzGerald et al., 2015). Importantly, the generative model predicts states that are consistent with an organism’s conditions for survival (i.e., those that are preferred; Parr et al., 2022; Smith, Friston & White, 2022). Surprising states (i.e., those that are not predicted by the generative model) thus become those that are not desired.

These predictions within the generative model are expressed mathematically as probability distributions (or ‘Bayesian beliefs’) that describe the likelihood of different sensory outcomes. These predictions are afforded different precisions (Friston et al., 2011). Precision captures the uncertainty associated with a prediction or sensory input, and corresponds to the inverse of the variance of the underlying probability distribution (for an example, compare the high precision proprioceptive prediction distribution with the low precision proprioceptive feedback distribution in the ‘Active inference’ box of Fig. 3). Precision weighting involves a zero-sum weighting in which higher precision is either afforded to top-down predictions or to bottom-up sensory input, depending on the level of uncertainty associated with each. This affects the extent to which sensory prediction errors are able to update predictions. Neurally, precision has been proposed to be encoded by synaptic gain (postsynaptic responsiveness) of superficial pyramidal cells in the cortex (Friston & Kiebel, 2009; Friston et al., 2011). Such changes in synaptic gain to implement changes in precision are proposed to be driven by both neuromodulators such as dopamine (Friston et al., 2011; Parr et al., 2022, 2025) and neural oscillations (Palmer et al., 2019; Parr & Friston, 2018; Sedley et al., 2016; for more detail of the neural implementation of these mechanisms, see Appendix 1).

During active inference, predictions (desires or goals) are afforded high precision and action thus seeks to achieve the sensory outcome that is predicted. Conversely, during perceptual inference, the predictions (beliefs) may be afforded different precisions, and the extent to which they are updated is determined by the relative precision of the prediction and the input. Precision may be modulated in a bottom-up fashion (e.g., based on the reliability of sensory feedback at a given moment) or in a top-down fashion according to current goals (i.e., whether an individual is listening or speaking). Indeed, to allow an organism to move at all, more precision must be afforded to proprioceptive predictions than feedback so that prediction errors are minimised through active inference (see high precision proprioceptive prediction in ‘Active inference’ in Fig. 3). We will illustrate this effect of changes in precision on speech behaviours in the context of responses to sensory perturbations in a later section (*An active inference account of compensation and adaptation*).

As highlighted by Yon et al. (2020) however, while this provides an explanation of how a single representation of prediction can in theory function as either a belief or a desire, this does not allow for the two to be represented simultaneously. That is, since the process of precision weighting is zero-sum, higher precision afforded to a prediction necessarily results in lower precision afforded to sensory feedback (and thus to prediction errors). This is troubling, since it appears to defy the intuition that it is possible for us to believe that one outcome is the most likely, but to desire another. It further suggests that perception is suspended during action, since higher precision predictions prevent the process of perceptual inference. That is, the same prediction cannot be used to represent both the belief as to the current location of an effector (with sensory feedback being used to perceptually monitor and update this prediction through error correction), and the desired movement goal (with sensory feedback and error correction being suppressed to ensure the prediction is not updated and the goal is fulfilled). However, we clearly do monitor the sensory consequences of our actions as they unfold, and engage in online error correction to ensure that our actions achieve their intended goals (Burnett et al., 1998; Desmurget & Grafton, 2000). The sufficiency of a single predictive representation in place of separate representations of beliefs and goals is thus called into question.

Such a challenge was answered by Smith, Ramstead, et al. (2022), who argued that beliefs and desires could be represented by different types of prediction errors in a hierarchical active inference model of decision making, in the way called for in Yon et al. (2020). While beliefs are represented by a lower-level ‘state’ prediction error (between the expected state and the current state) that can drive perceptual belief updating (and allow us to perceive the sensory outcomes of our actions while moving), desires are represented by a higher-level ‘outcome’ prediction error (between the expected outcome of a particular action and a ‘desired’ outcome) which drives the intention to act. This type of hierarchical structure with prediction errors at different levels of abstraction shows some parallels to the FACTS model of speech motor control (Kim et al., 2023; Parrell, Ramanarayanan, et al., 2019), an extension of the SFC model which also incorporates a distinction between higher level desired task states (defined as constrictions of the vocal tract) and lower level articulatory state estimates (beliefs about positions of individual articulators). This model will be discussed further in a later section (*Speech motor control model accounts of compensation and adaptation*).

### The perception–action relationship

Active inference is distinct from traditional models of speech motor control in that it aims to provide a unified mechanistic understanding of both perception and action. That is, in an active inference account the same generative model is used to generate action, and to perceive the sensory consequences of an action produced by other agents (Friston et al., 2011; Pickering & Clark, 2014). This is achieved by manipulating the amount of precision afforded to proprioceptive signals. During observation of others’ actions, we generate all the same predictions as we would during performance of that action ourselves (including proprioceptive predictions). The absence of proprioceptive feedback would thus have the potential to generate large proprioceptive prediction errors; however, by down-weighting the precision of proprioceptive predictions, these prediction errors are afforded very low precision. This means that the capacity of prediction errors to drive either active inference (i.e., to generate movement) or perceptual inference (i.e., to update proprioceptive predictions) is effectively switched off. Conversely, other types of sensory prediction errors (e.g., visual or auditory) can be used to update predictions via perceptual inference. The system is thus able to use all other aspects of the generative model to infer the hidden state (i.e., the action or intention of the other agent) without engaging in movement (i.e., in minimisation of proprioceptive prediction errors via active inference).

The idea of shared mechanisms for perception and production is not new in the field of speech research. One influential and intensely debated attempt to equate the two processes is to be found in the motor theory of speech perception (Liberman & Mattingly, 1985), which originally proposed that motor representations (i.e., speech articulatory gestures) are the targets of speech perception. Even opponents of these views now advance a more nuanced account, in which motor areas can play a supporting role in speech perception under more challenging listening conditions, such as background noise (Stokes et al., 2019; Wilson, 2009; Wu et al., 2014). By contrast, active inference turns this idea on its head to present an idea that is equally as radical—namely, that an equivalence between motor and sensory systems is not to be found in the role of the motor system in perception, but rather in the sensory nature of motor commands. Other accounts similarly propose a common mechanism of prediction across action and perception in speech, without however invoking the more radical claim that motor commands are themselves simply sensory predictions (Gauvin & Hartsuiker, 2020; Pickering & Gambi, 2018; Pickering & Garrod, 2013; Skipper et al., 2017). For example, Pickering and Garrod (2013) propose that the same forward models are employed for prediction of speech produced by both the self and by others, across multiple levels of linguistic representation (i.e., across phonology, semantics, and syntax).

Conversely, the traditional computational models of speech motor control reviewed here only address the processing of self-produced speech feedback during speech production; mechanisms for perception or recognition of speech produced by others are outside their scope. A role for perception of other voices is included in the DIVA model during the development of speech production, with input from other speakers being used to learn and refine sensory (auditory) target regions for speech sounds in the infant’s native language (Guenther, 2016). However, this part of the model does not explain how or why auditory targets continue to be influenced by sensory input from other talkers across the lifespan. In contrast, active inference accounts for this continued influence of perceived speech on production by proposing that the auditory predictions that are updated through perceptual inference during perception of other’s speech are the very same auditory predictions that drive active inference in generating one’s own speech.

### Summary of model comparisons

Overall, all three models share the common central tenet that sensory prediction errors can be minimised through action, as well as through prediction updating. However, the models differ in the exact nature of the pathways through which such prediction error minimisation can occur. Interestingly, active inference appears to share common elements with both preexisting speech motor control models: (1) the exclusive reliance on a ‘feedback-control-like’ mechanism for action generation places active inference closer to SFC, while (2) the inclusion of two routes for sensory prediction error minimisation (perceptual inference versus active inference) more strongly parallels DIVA’s distinction between offline updating of stored forward models (blue arrow on Fig. 2A) versus online correction of movement (green arrow on Fig. 2A). Active inference differs from both models however in that it seeks to provide an integrated account of both action and perception (of the self and others).

The following section will explore to what extent these differences between models result in different predictions for sensorimotor behaviours in speech, and review existing empirical evidence that may help adjudicate between them.

## Behavioural phenomena in sensorimotor control of speech

### The sensory perturbation paradigm

**Fig. 4:**
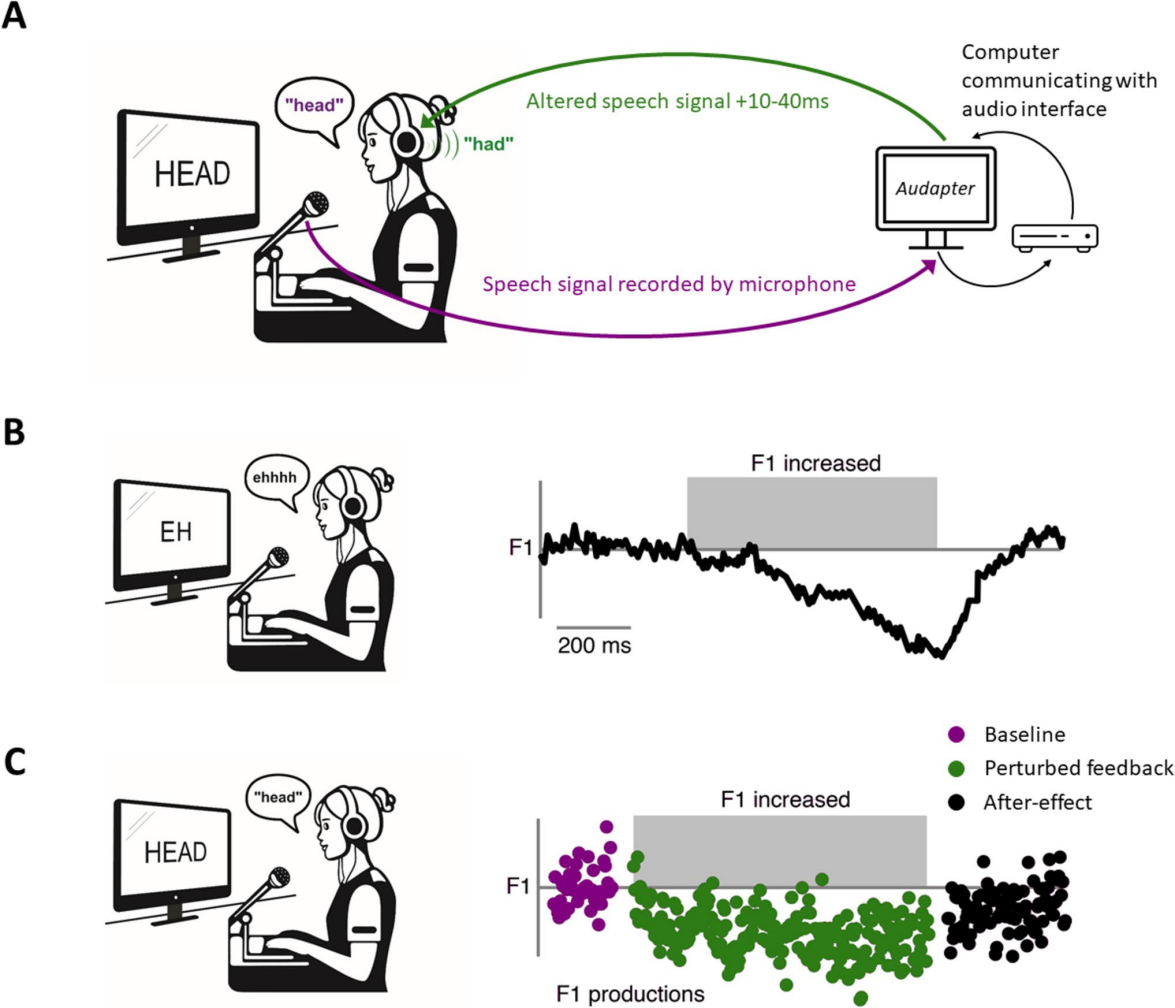
The altered auditory feedback paradigm. **A** Schematic illustrating the set-up for an altered auditory feedback experiment. Participants typically read aloud single words whilst hearing their own speech played back via headphones in near real-time, with or without alteration (e.g., an upward shift of the first formant makes the / ɛ/ vowel in head sound more like the /æ/ vowel in had). This is commonly implemented using the software programme Audapter (Cai, 2015) which performs real-time signal processing via an audio interface. **B** Example dataset showing compensation to a random perturbation of the first formant (F1, the lowest frequency resonance of the vocal tract during speech), adapted from (Burnett et al., 1998). Line graph shows changes in produced F1 across time within a single utterance of the vowel sound /ɛ/ as in ‘head’. Within a few hundred milliseconds of hearing their voice with an upwardly-perturbed F1, participants compensate by lowering F1 in their speech. **C** Example dataset showing adaptation to a sustained auditory perturbation of F1, adapted from (Lametti et al., 2018). Dots indicate F1 frequency as produced by a single individual during repeated utterances of the word ‘head’. These experiments typically begin with a baseline period in which auditory feedback is unaltered (purple dots), followed by a perturbation period in which an alteration of auditory feedback (an increase in F1 in this example) is applied consistently over trials (green dots). After tens of trials of hearing an increased F1, participants adapt by subsequently producing vowels with a lower formant frequency. This adaptation can persist in trials immediately after the cessation of formant perturbation (the after-effect phase, black dots). (Colour figure online)

A key method for investigating error correction during speech production is via sensory perturbation paradigms, such as the altered auditory feedback paradigm (Houde & Jordan, 1998). Here, a speaker’s speech auditory feedback (i.e., the sound of their voice as they are speaking) is recorded by a microphone and played back in perturbed form via headphones in near real-time (with <50-ms delay; see Fig. 4A). This alteration typically involves a change to the acoustic and spectral properties of the speech, such as its fundamental frequency (F0, the acoustic correlate of pitch) and formant frequencies (vocal resonances that signal vowel identity). Speakers typically oppose the direction of the perturbation by making unconscious compensatory adjustments to their productions (e.g., by lowering pitch in response to an upward pitch shift). This effect suggests a system that compares auditory feedback with top-down predictions, and aims to minimise error between the two.

Perturbed feedback can either be presented randomly on an unpredictable number of trials (Burnett et al., 1998), or be sustained across repeated utterances (Houde & Jordan, 1998). In the unpredictable case, participants show online alterations to production within the time-course of single syllables (typically termed ‘compensation’), which do not persist once the feedback alteration is removed (see Fig. 4B). Conversely, in the sustained case, compensatory changes increase across trials—a phenomenon known as speech motor adaptation (see Fig. 4C). Intriguingly, in contrast with the random perturbations, these changes persist after the perturbation is removed (Purcell & Munhall, 2006). These after-effects suggest some level of offline sensorimotor updating has occurred.

### Speech motor control model accounts of compensation and adaptation

The DIVA model attributes these compensation and adaptation effects to the operation of feedback and feedforward control systems respectively, which provide two routes for prediction error minimisation. Unpredictable or inconsistent errors in sensory feedback trigger online corrective adjustments to the motor programme by the feedback controller (see green arrow in Fig. 2A). Crucially, these rapid online corrections occur *in the absence* of changes to forward model predictions (and the synaptic weights that encode them), accounting for the short-lived nature of compensation responses. Conversely, when these errors are consistently detected over time, the online adjustments to the motor commands issued by the feedback controller become integrated into the motor commands sent by the feedforward controller (involving changes to internal models and underlying synaptic mappings) via a slower offline learning process (blue arrow in Fig. 2A), ensuring that these targets remain up to date and accurate. This explanation accounts for the persistence of adaptation responses after the feedback alteration is removed. In this way, DIVA allows for auditory prediction errors to be resolved either directly through online modifications to an ongoing action, or indirectly through offline updating of stored feedforward motor commands.

By contrast, SFC does not include a direct route by which auditory prediction errors can be used to drive action; instead, auditory prediction errors have their effect on action indirectly, by updating the state estimate of the articulators and the state prediction (see blue arrows in Fig. 2B), which in turn affect the generation of speech motor commands by the feedback control system. This is the pathway proposed to underlie the compensation response. To model the adaptation response however, an extension of the original SFC framework has been proposed in the FACTS model (Kim et al., 2023). This takes the existing lower-level articulatory state estimator architecture in SFC (concerned with positions of individual articulators), and duplicates it at a higher order ‘task’ level (concerned with constrictions of the vocal tract; e.g., the extent of constriction between the tongue body and palate). Importantly, the system learns a forward model mapping between the lower-level articulatory states and the higher-level task states. During adaptation, consistent auditory prediction errors are used to update the task estimate (i.e., beliefs about the current constriction of the vocal tract) which in turn updates this forward model mapping. This new mapping can then be used to generate articulatory motor commands that will minimise the task-level prediction error, and bring the task state estimate back in line with the task state target (i.e., the desired vocal tract constriction). The crucial distinction between adaptation and compensation according to FACTS therefore lies in the presence or absence of this forward model updating process.

### An active inference account of compensation and adaptation

To our knowledge, active inference has not previously been applied to explain compensation and adaptation in speech motor control; we therefore present here an initial account of these behaviours based on active inference principles. Active inference assumes that sensory prediction errors can drive changes to action by updating proprioceptive predictions (see middle blue arrow in Fig. 2). This is an important implication of the claim that ‘motor commands’ are themselves sensory (proprioceptive) predictions. That is, to correct for an auditory perturbation, proprioceptive predictions must be updated to bring about prediction errors that can then be suppressed through classical reflex arcs to generate the (corrective) movement. In this sense, corrections for auditory errors in speech (even those that are random) must always trigger prediction updating in the proprioceptive domain for a new movement trajectory to be implemented.

An active inference account of the developmental acquisition of speech by Najnin and Banerjee (2017) demonstrates how auditory prediction errors can be minimised through action by driving proprioceptive prediction updating. Here, production of a speech sound is achieved through ‘inversion’ of a generative model that specifies mappings between proprioceptive causal states and auditory outcomes. This inversion allows the reverse mapping to be used to translate a desired auditory target into its associated proprioceptive causal state (i.e., a configuration of the articulators that is expected to cause the appropriate speech sound to be produced). The resulting proprioceptive prediction error can then be minimised through active inference to achieve this proprioceptive prediction and produce the speech sound (assuming that the proprioceptive to auditory mapping is sufficiently accurate). During speech acquisition, the infant must refine this generative model using auditory feedback to learn the mappings between proprioceptive states and auditory outcomes.

**Fig. 5:**
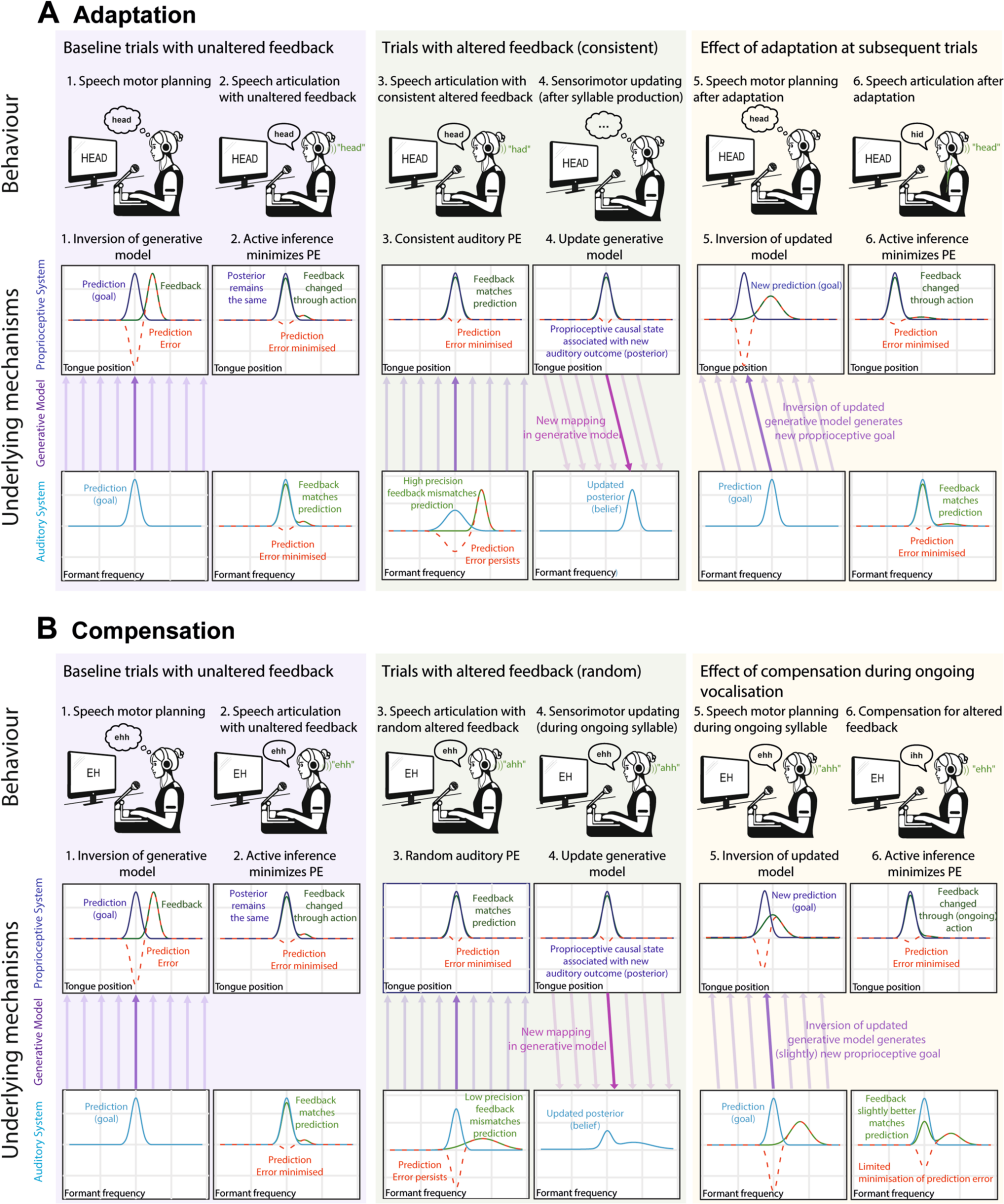
Active inference mechanisms for (**A**) adaptation, (**B**) compensation. Paired plots illustrate prior predictions (blue), sensory feedback (green), prediction errors (red), posterior (updated) predictions (blue) as probability distributions, for proprioceptive (top) or auditory representations (bottom). The x-axes show tongue position and formant frequency: dimensions for which proprioceptive and auditory predictions are compared to sensory feedback. Precision is reflected in the variance of these distributions (larger variance indicating reduced precision, increased uncertainty). Numbered headings (1–6) represent time points during a trial sequence (adaptation, **A**), or single trial (compensation,** B**), grouped into three stages: Baseline, purple; Altered feedback, green; After adaptation/compensation, yellow boxes. (Colour figure online)

Figure 5 translates this process into the context of auditory feedback perturbations in the mature system. A step-by-step description of these processes (Time Points 1–6 in Fig. 5) is given in the figure caption. In summary, auditory perturbations mean that the proprioceptive target initially inferred from the auditory target (through inversion of the generative model) no longer achieves the expected auditory outcome—that is, results in auditory prediction error (see Time Point 3 in Fig. 5). This auditory prediction error leads to updating of the predicted auditory outcome that is associated with the current proprioceptive state (through perceptual inference; see middle blue arrows intersecting with purple arrows in the generative model on Fig. 3), and this updated mapping is generalised across the generative model (see pink arrows in Time Point 4 of Fig. 5). This updated generative model is then inverted once again using the original auditory target to infer a new proprioceptive target that should now achieve an auditory outcome that more closely matches that predicted/intended (see Time Points 5 and 6 of Fig. 5). It should be noted, therefore, that while this process does involve updating an auditory prediction, here the prediction is acting as a *belief* (as to what auditory consequences are associated with different proprioceptive sensations); conversely, the auditory prediction corresponding to the intended auditory *goal* remains unchanged (as can be seen in Time Point 5 of Fig. 5).

Crucially, the extent of this updating of the generative model will depend on the relative weighting of precision afforded to auditory feedback versus the auditory prediction. Random perturbations that vary from utterance to utterance will cause auditory feedback to be afforded less precision relative to the prediction, since auditory feedback appears unreliable (see Time Point 3 of Fig. 5B). Such precision weighting means that the prediction is more resistant to updating (i.e., the posterior distribution is shifted less relative to the prior), resulting in more limited updating of the generative model (see Time Point 4 of Fig. 5B). Inversion of this (slightly updated) generative model during ongoing vocalisation thus still yields some auditory prediction error (as in Time Point 6 of Fig. 5B), albeit less than before. Conversely, consistent perturbations will yield higher precision for auditory feedback relative to the prediction (see Time Point 3 of Fig. 5A), allowing greater updating of the generative model and thus a greater reduction of auditory prediction error on subsequent utterances (see Time Point 6 of Fig. 5A).

Ultimately therefore, active inference assumes that both adaptation and compensation rely on the same mechanism (i.e., the basic processes outlined in Fig. 5A and B are the same). The difference between these behaviours is thus reduced from a qualitative to a quantitative distinction: they differ only in the level of precision afforded to sensory feedback, and thus the extent to which the generative model (and accordingly the inferred proprioceptive target) is updated (compare Time Point 4 of Figs. 5A and 4B). This accords with empirical findings that the compensation response is typically of smaller magnitude than the adaptation response (Raharjo et al., 2021).

1. Inversion of generative model. During speech motor planning, inversion of the generative model (purple arrows) allows a proprioceptive target (dark blue distribution) to be inferred from specific auditory targets (light blue distribution). Proprioceptive feedback and prediction error distributions reflect the resting position of the articulators.
2. Active inference minimises prediction error. Proprioceptive prediction error is minimised through active inference (compare 1, 2). If auditory feedback is unaltered (generative model mappings are accurate) then minimising proprioceptive prediction error reduces auditory prediction error and correct speech sounds are produced.
3. Auditory perturbation leads to persistent prediction error. When auditory feedback is perturbed minimisation of proprioceptive prediction error results in auditory feedback that mismatches with predictions (compare 2, 3), and persistent auditory prediction error*.*
4. Updating generative model. Prediction error drives updates to the generative model: Auditory prediction error is minimised by changing the auditory predictions (blue) associated with the current proprioceptive state (pink arrows). The extent of updating depends on the precision of auditory feedback. For adaptation (A4), consistent perturbation gives high precision auditory feedback and low precision auditory predictions, hence greater updating of the generative model (pink arrows). For compensation (B4), random trial-wise perturbations lead to low precision of auditory feedback and higher precision auditory predictions, hence less updating of the generative model (compare A4, B4).
5. Inversion of updated model. Inversion of the updated generative model using the original auditory target (after adaptation, (A)) or during compensation (B)) yields modified proprioceptive predictions (shifted blue distributions).
6. Active inference minimises prediction error (PE). Active inference to enact this new proprioceptive target results in reduced auditory prediction error in produced speech. Auditory prediction error persists after compensation (B) due to incomplete updating of the generative model. (Colour figure online)

In this way, active inference is like SFC/FACTS in that these models also predict that motor correction of prediction errors in auditory feedback will always be accompanied by updating of predictions concerning the state of the articulators/vocal tract. However, these models differ from an active inference account in that this prediction updating means that the representation of the current articulatory/task state no longer matches reality (i.e., the new prediction corresponds to a belief that the articulators are in a different state than they actually are). Conversely, in the account proposed here, the belief that is updated corresponds to the auditory outcome associated with the current proprioceptive state (itself directly signalled by proprioceptive feedback) in the generative model. Further, while SFC/FACTS attribute compensation to prediction updating (of the articulatory state estimate/prediction), this is distinct from the process underlying adaptation, in which the articulatory-to-task transformation (forward model) is updated. The current active inference account is thus unique in predicting that some (albeit limited) updating of forward model-like predictions (i.e., transformations from proprioceptive states to auditory outcomes) will be apparent even in response to random perturbations.

Some evidence that compensation does indeed involve changes to forward model predictions was recently reported in a study by Hantzsch et al. (2022), who demonstrated that prediction updating can occur even after a single exposure to auditory error. Specifically, by combining data from across multiple studies using random auditory perturbations (involving >90 participants), they demonstrated that speakers showed carry-over of compensation on unperturbed trials that were immediately preceded by perturbed trials. This suggests that even random and unreliable auditory prediction errors trigger some updating of forward-model predictions, but to a lesser extent than for reliable prediction errors. This finding thus supports the idea that compensation and adaptation both operate through a shared mechanism of prediction updating, with only a quantitative distinction between them. However, this result is (arguably) incompatible with both the DIVA and SFC/FACTS frameworks, which assume that consistency in prediction errors and/or compensatory adjustments is required in order for forward model updates to occur (i.e., for feedback corrective commands to be incorporated into the feedforward controller as in DIVA, or for forward model mappings between the lower-level articulatory states and the higher-level task states to be updated as in FACTS).

Overall, therefore, while DIVA and SFC assume a qualitative distinction between the mechanisms underlying compensation and adaptation (e.g., with differences in the involvement of forward model updating), active inference collapses this into a quantitative distinction in terms of a probabilistic reweighting of precision. At the neural level, motor adaptation (e.g., for arm movements) has been shown to involve long-term synaptic plasticity, with lasting functional neural changes (i.e., changes to the tuning properties of individual neurons) accompanied by structural changes (e.g., generation of new dendritic spines, new axonal collaterals, and changes to myelination; Della-Maggiore et al., 2015). Conversely, rapid compensation responses to unpredictable sensory errors are conventionally assumed not to involve these synaptic plasticity mechanisms. Indeed, as previously outlined, both DIVA and SFC/FACTS accounts assume no updating of sensorimotor mappings (i.e., forward models) during compensation, and thus, presumably, no associated changes to synaptic weights via plasticity mechanisms. For example, a recent simulation of the reflexive pitch shift response within the ‘simple DIVA’ framework, a simplified version of the DIVA model, found that online compensation to unexpected pitch shifts could be simulated by just three parameters; the gain of the auditory feedback controller’s response to a perceived error, the delay of this response, and the gain of the somatosensory feedback controller (Kearney et al., 2022).

However, elsewhere these authors have acknowledged that direct testing of claims regarding the absence of synaptic plasticity for compensation is difficult (or sometimes impossible), given the challenges involved in measuring characteristics such as the number, strength and plasticity of synapses in vivo in humans (Kearney et al., 2020). Outside of speech, recent proposals have challenged the view that synaptic plasticity is exclusively for motor learning and not for online compensation; for example, simulations of cerebellar circuits using a computational model of ‘Bayesian plasticity’ have been used to argue for the involvement of rapid changes to synaptic weights in driving immediate compensation for sensory errors, complemented by a slower plasticity process that implements longer-term adjustments to synaptic weights for learning (Bicknell & Latham, 2025). The active inference account of compensation and adaptation proposed here would suggest that changes in precision would be accompanied by fast changes in synaptic weights driven by neuromodulators such as dopamine, potentially working in concert with changes in neural oscillations (see Appendix 1). Any resulting changes to the generative model would be further expressed as changes to synaptic weights; however, in the context of unexpected perturbations, such changes would be small and short lived, making their detection difficult. Testing these differing predictions of the models in the context of neural data is therefore challenging. Existing behavioural evidence of ‘after-effects’ following compensation to random perturbations (i.e., ‘one-shot adaptation’; Hantzsch et al., 2022; Ruttle et al., 2021) provides one piece of evidence for adjudicating between these views.

### Detection and correction of auditory errors

The question of whether sensory predictions are derived from desired targets (as in DIVA) or beliefs about the current state of the articulators (as in SFC) has important implications for how and when auditory feedback perturbations are corrected. SFC predicts that compensation occurs in response to any perturbation of auditory feedback (since this results in a deviation from efference-copy based beliefs); conversely, DIVA predicts compensation only for perturbations that push sensory feedback outside of a target/goal region representing the desired sensory outcome. Interestingly, the same magnitude of auditory feedback perturbation has been shown to induce greater compensation when it pushes feedback across an individual’s perceptual category-boundary compared with when it does not (consistent with DIVA); however, within-category perturbations were nevertheless still compensated for (consistent with SFC; Niziolek & Guenther, 2013). Neither DIVA nor SFC alone therefore capture this combined pattern well.

**Fig. 6:**
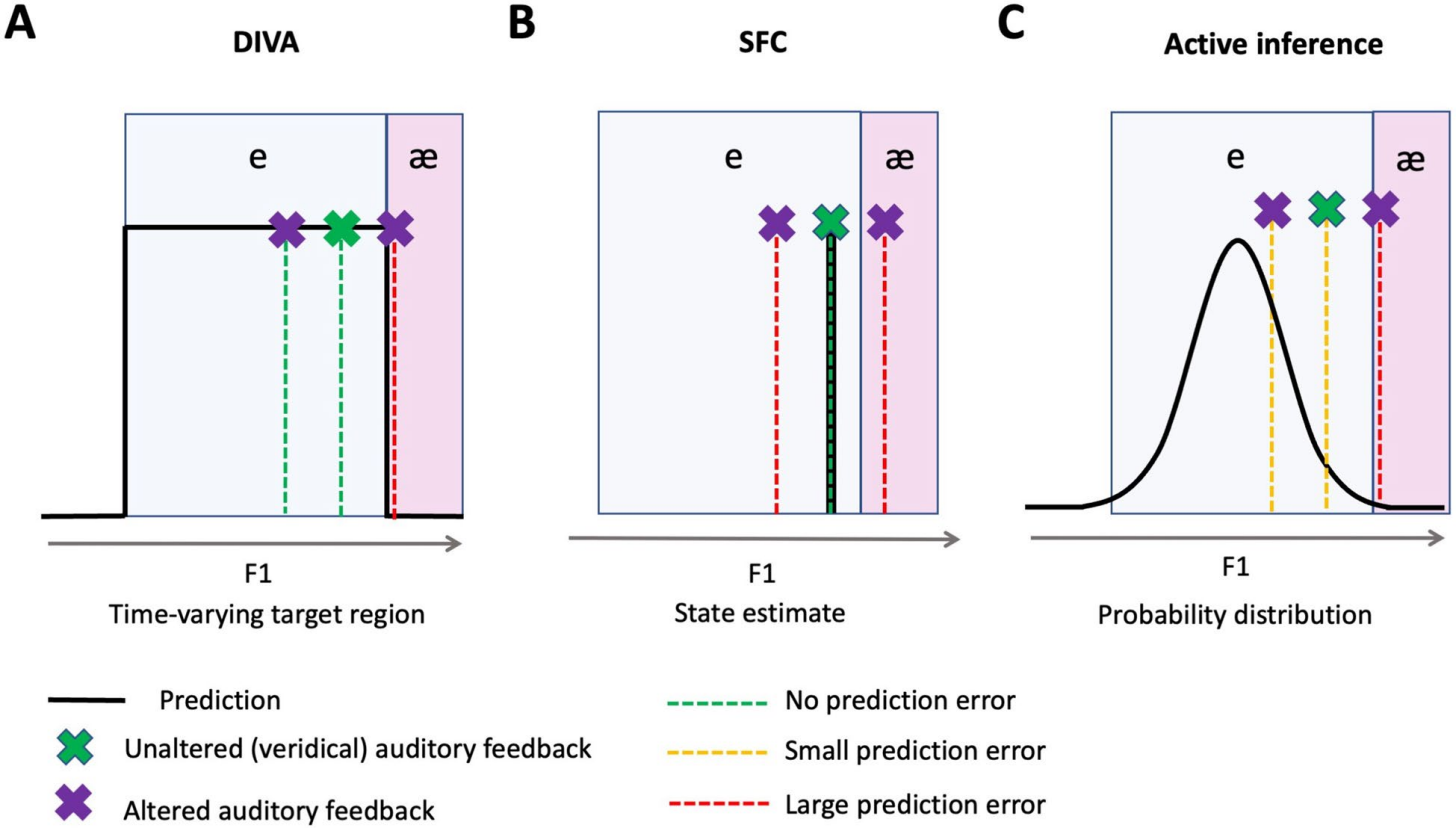
Predictions of the models for responses to within- and between-category perturbations of sensory feedback.** A** DIVA implements sensory predictions as a target region; this means that a perturbation of the same magnitude will generate different prediction errors depending on if it crosses a category boundary or not. The model is thus insensitive to perturbations that keep sensory feedback within the target region, since no prediction error is generated. **B** SFC implements sensory predictions as a precise expected end state, based on an efference-copy of the motor commands that were sent out. This means that SFC would expect equal prediction errors for a perturbation that moves feedback across a category boundary, and a perturbation of equal magnitude that keeps feedback within the same category. **C** Active inference implements sensory predictions as probability distributions that could be bounded by category boundaries; this means that perturbations within the bounds of the probability distribution will still be detectable (i.e., generate a prediction error), but will result in smaller prediction error than a between-category perturbation. This also has the implication that less prototypical productions (even in the absence of ‘altered feedback’) will induce a small prediction error (see green cross). (Colour figure online)

An active inference account of speech motor control may be better able to explain this outcome, since in active inference sensory predictions take the form of probability distributions, with a mean and variance (precision). In the case of speech, the bounds of the probability distribution representing the auditory sensations predicted for a given speech sound (i.e., a phoneme) can be considered to correspond to the boundaries for that phoneme category. Changes in the precision of auditory predictions will thus have consequences for the level of acoustic variation that is tolerated for a given phoneme category. This added complexity allows a certain level of sensitivity to sensory errors that deviate from the centre of a sensory probability distribution, but are nevertheless still contained within that distribution, while also predicting greater prediction error for deviations of the same magnitude that push sensory feedback beyond the limits of the probability distribution. In this way, rather than prediction error being ‘all-or-nothing’ as in DIVA (i.e., sensory feedback is either outside the target-region or inside it), or entirely graded around a very precise prediction as in SFC, active inference combines the two such that prediction error is graded across a confined probability distribution (see Fig. 6). This probabilistic aspect of active inference is thus better able to account for findings concerning within- versus between-category perturbations. It should be noted, however, that this aspect is shared by other Bayesian modelling accounts (e.g., (Patri et al., 2018, 2019). A further interesting implication is that productions that naturally fall towards the tails of this distribution will generate prediction error; this accords with evidence for within-utterance corrective movements for less prototypical vowel productions in the absence of altered feedback (Niziolek et al., 2013).

### Perception and production interactions in speech: Phonetic convergence

As we introduced previously, active inference accounts propose that the same generative model is used both to generate action and to perceive the sensory consequences of actions produced by other agents. As such, it leads us to expect bidirectional interactions between the processes of action and perception. The domain of speech offers numerous examples in which perception and production processes exert significant influences over one another. We have already seen in the previous section that phoneme category boundaries in passive perception have implications for responses to acoustic perturbations during production of those same speech sounds (Niziolek & Guenther, 2013), suggesting the shared use of category boundaries across production and perception. This is directly predicted by active inference, which assumes that the same set of probability distributions serve as predictions across action and perception.

A further relevant phenomenon for action-perception interaction in speech is that of phonetic convergence (also referred to as alignment, accommodation or entrainment), in which the voices of two speakers tend to become more similar to one another in terms of their acoustics across the course of an interaction (Aubanel & Nguyen, 2020; Babel, 2012; Bradshaw & McGettigan, 2021; Goldinger, 1998; Pardo, 2006; Pardo et al., 2012, among many others). Traditionally such effects have been studied and explained from a social psychology perspective (Giles et al., 1991), with findings suggesting the influence of social factors such as gender, attractiveness, likeability and perceived social status on convergence behaviour (Bourhis & Giles, 1977; Gregory & Webster, 1996; Michalsky & Schoormann, 2017; Namy et al., 2002). More recently however it has been suggested that this convergence may also (at least in part) reflect the operation of a lower-level sensorimotor learning process (Sato et al., 2013; Späth et al., 2022). If so, this would allow for conceptualisation of phonetic convergence within the same mechanistic framework as speech motor adaptation; that is, as arising from a common mechanism of prediction error minimisation.

Framing these two behaviours as reflecting a common underlying sensorimotor learning process however appears problematic, given differences in the direction of the two responses. In adaptation, prediction error between an internal target and auditory feedback is minimised through driving changes to speech productions in an opposing direction to the error. Conversely, in convergence, ‘prediction error’ between auditory input from the self-voice and the other voice is minimised through driving changes in the same direction as the error, towards the other voice. We are therefore left with the question of why and how the brain seems to respond to sensory errors differently in the two cases.

We propose that an active inference theory offers a potential resolution of this paradox. Active inference naturally conceives of these two processes as operating on a common set of sensorimotor predictions and a unified process of prediction error minimisation. These sensorimotor predictions can be modified by both speech sensory input from the self-voice (as in adaptation), and from other voices (as in convergence). Crucially, precision weighting and the distinction between perceptual and active inference may offer a solution to apparent differences in the direction of these responses. During speaking, active inference requires that predictions are afforded higher precision than sensory evidence. Prediction errors induced by perturbations of self-voice feedback during speaking thus do not trigger updating of predictions, but are resolved through corrective changes to action (speech movements) to force sensory feedback to conform to predictions (i.e., the acoustic target for the speech sound). This results in a response that moves the voice in the opposite direction to the error.

Conversely, when listening to another speaker, perceptual inference requires higher precision to be afforded to sensory evidence than to sensory predictions (which are likely to be uncertain, particularly when listening to an unfamiliar speaker). Prediction errors are thus minimised by changing predictions to fit the sensory input. This results in updating of the generative model to express a modified ‘belief’ as to how a particular speech sound should be realised acoustically, to be closer to the acoustic realisation produced by the other speaker. Phonetic convergence would then ensue when the listener uses this updated prediction as a target for their own subsequent speech production (through active inference). This results in a response that seemingly moves the voice in the same direction as the sensory error. In this way, these apparent conflicting responses are reduced to being two-sides of the same coin.

Such an account may also be applicable for understanding individual variability in responses to auditory feedback perturbations. While responses at the group level typically oppose the direction of the perturbation, at the individual level so called ‘following responses’ are frequently reported, where speakers instead shift their speech in the same direction as the perturbation (Miller et al., 2023). While the underlying mechanism is still debated (Franken et al., 2018; Miller et al., 2023), one dominant account proposes that they reflect attribution of altered feedback to an externally generated source (i.e., another speaker), rather than being recognised as an error in self-generated speech (Franken et al., 2023; Hain et al., 2000; Patel et al., 2014). An active inference account would expect changes in attribution of the source of the altered feedback to be accompanied by changes in the precision afforded to auditory input as outlined above (i.e., lower precision auditory predictions are made for other voices than for the self-voice). Following responses would thus ensue via the same mechanism as phonetic convergence—that is, via an updating of auditory predictions (through perceptual inference) that are then used to drive one’s own productions. Changes in precision weighting however can be brought about by multiple different top-down and bottom-up factors, and thus this account need not always necessitate a change in agency for following to occur. The resulting conceptualisation of opposing and following responses as two extremes of a continuum—with changes in precision of auditory feedback allowing a smooth gradation between the two—may align better with recent meta-analysis findings that responses to altered auditory feedback form a unimodal distribution, arguing against a qualitative distinction between the two response patterns (Miller et al., 2023). 

The proposal that a shared set of sensorimotor predictions operate during both phonetic convergence and speech motor adaptation is supported by evidence from a series of studies using synchronous speech tasks with real-time perturbations of auditory feedback, to investigate interactions between the two processes (Bradshaw et al., 2023, 2024). Synchronous speech or ‘choral speech’ refers to the act of speaking in synchrony with another speaker, and has been shown to induce convergence in speech acoustics (e.g., pitch) between pairs of speakers, besides the obvious convergence in their speech timing (Bradshaw & McGettigan, 2021). By employing real-time formant perturbations to both a speaker’s own voice and their synchronisation partner’s voice during the task, Bradshaw and colleagues manipulated the congruency of the direction of formant change required for simultaneous convergence and adaptation. When formant changes that would be driven by these two processes were in conflict with each other, reduced adaptation was observed, compared with a group for which these formant changes were in agreement (Bradshaw et al., 2023, 2024). We propose that this pattern of results is best explained by assuming that phonetic convergence involves an updating of the same sensory (auditory) predictions that are used for sensorimotor learning with the self-voice. While these findings are entirely in line with our active inference theory, no such effects would be predicted for an account in which convergence and adaptation involve separate mechanisms.

In addition to these phonetic convergence effects, other studies have shown bidirectional interactions between speech motor adaptation and perception of other voices. Speech motor adaptation to formant perturbations has been reported to be moderated by explicit perceptual training that results in a shift in the perceptual category boundary between two vowel sounds (Lametti, Krol, et al., 2014a, 2014b). Changes to adaptation also arise after implicit perceptual learning processes; mere exposure to a voice with altered formants has been found to affect subsequent correction for formant perturbations in one’s own voice (Bourguignon et al., 2016). On the reverse side, speech motor adaptation has also been shown to alter perception of other voices, causing a shift in phoneme category boundaries (Lametti, Rochet-Capellan, et al., 2014a, 2014b; Shiller et al., 2009). Modelling work by Patri et al. (2018) using the GEPPETO model framework, a Bayesian model of speech communication, demonstrates how this phenomenon can be accounted for by assuming shared acoustic targets across production and perception, and involvement of somatosensory pathways in perception. Both assumptions are also embodied by our active inference account.

These findings thus point towards the use of a shared set of predictions underlying both perception of other voices and sensorimotor control of the self-voice. This assumption is central to an active inference perspective but absent from traditional models such as DIVA and SFC that are focused solely on speech motor control. We acknowledge however that phonetic convergence is complex and often context dependent. It is not limited to lower-level sensorimotor processes but is influenced also by a broad range of social, linguistic, perceptual and cognitive factors (e.g., Kim & Clayards, 2019; Michalsky & Schoormann, 2017; Nielsen, 2011; Pardo et al., 2017). A fully comprehensive account of phonetic convergence must therefore capture these multiple interacting levels. Active inference and predictive coding theories have been applied to various levels and domains of functioning, from lower-level sensory perception (Clark, 2013) to higher-level social and communicative processes during interpersonal interactions (Bouizegarene et al., 2024; Friston et al., 2020; Jiang et al., 2021). These frameworks therefore offer the potential for a unified mechanistic account of speech behaviours such as phonetic convergence, that are embedded across these different levels of processing. It will be for future experimental and computational modelling research to flesh out this active inference proposal.

## Summary and future directions

Throughout this review, we have aimed to illustrate how predictive coding and active inference can be readily applied to the study of speech motor control, starting with a focus on auditorily guided speech production. We have presented proposals for how such an account might explain otherwise challenging findings concerning correction for auditory perturbations. As we have illustrated, active inference has much in common with the two models of speech motor control covered in this review. However, it also provides a novel perspective on the following three aspects of speech motor control, leading to new explanations of existing research and new predictions for future research:

1. Active inference recasts the relationship between compensation and adaptation responses to auditory feedback perturbations, reducing the distinction between them to a quantitative one in terms of the level of precision afforded to auditory feedback versus predictions. In so doing, it is able to account for one-shot adaptation after a single exposure to altered auditory feedback (Hantzsch et al., 2022), something that current models are less able to capture. Combined neuroimaging and computational modelling work can provide further tests of this claim; for example, we might observe neural correlates of changes in precision weighting (such as differences in the levels of specific neuromodulators or in the power of neural oscillations) following exposure to random versus sustained perturbation paradigms. Random perturbations should lead to reduced precision of sensory signals and prediction errors with implications for the rate at which updates are made to generative models. We can test for causal effects of precision weighting by using pharmacological or brain stimulation manipulations which should—by active inference theories—lead to changes in the magnitude of compensation and/or the rate of sensorimotor adaptation. The effects of brain stimulation on responses to sensory perturbations during speaking have been somewhat inconsistent across studies (Demirel et al., 2025; Lametti et al., 2018; T. L. Scott et al., 2020; Shum et al., 2011; Tang et al., 2021). Computational models based on active inference would allow for estimation of parameters such as precision within these experiments, which could be related to neural activity in the presence and absence of stimulation; this may provide better insights into the mechanisms underlying the effects of different stimulation methods on speech sensorimotor control.
2. By encoding predictions as probability distributions, active inference can account for reduced (but intact) sensitivity to perturbations that keep speech productions within the same phoneme category as compared to between-category perturbations (Niziolek & Guenther, 2013). This is in contrast with existing accounts. By reframing motor commands as proprioceptive predictions, active inference also provides a biologically plausible mechanism by which auditory prediction errors can be mapped to changes in action via an updating of proprioceptive sensory predictions. Precision weighting further provides a mechanism which allows the relative influence of sensory predictions versus sensory feedback to be flexibly modulated. Future work could test these mechanisms in the context of speech by exploring the effect of sensory degradation on correction for sensory errors in different modalities, as has been done in other domains (Chancel & Ehrsson, 2023; Limanowski & Friston, 2020). For example, ongoing work from our group is exploring the impact of spectral degradation of speech auditory feedback on sensorimotor speech learning (Bradshaw et al., 2025). Other work has shown the impact of somatosensory degradation through the application of topical oral anaesthesia on speech intelligibility (De Letter et al., 2020). Active inference accounts would predict that these and other manipulations that reduce the precision of sensory feedback will impact sensorimotor learning for speech.
3. By assuming a shared set of predictions across action and perception, active inference is readily able to account for bidirectional interactions between perception of other people’s voices and speech motor control. In particular, it can resolve an apparent paradox concerning the distinction between error correction responses observed in speech motor adaptation and phonetic convergence. Existing models such as DIVA and SFC currently cannot accommodate both these effects since phonetic convergence depends on mechanisms operating during speech perception, which are outside their scope. This limits the use of DIVA and SFC in understanding real-life speech motor control during multitalker interactions, where simultaneous adaptation of the self-voice and convergence with other voices is shown by recent results (Bradshaw et al., 2023, 2024). In brain imaging, active inference would lead us to expect shared neural correlates of predictive processing during speech production and perception. For example, active inference hypothesises a shared neural code for prediction errors during speaking and listening in speech-responsive auditory areas (e.g., superior temporal gyrus). Recent fMRI studies have failed to observe cross-decoding of heard and produced vowels in auditory regions using multivariate decoding techniques (Rampinini et al., 2017), perhaps because their listening and speaking conditions were not matched for expectedness (and hence prediction error). Showing cross-decoding between listening and speaking conditions that are equated for prediction strength would provide evidence in line with an active inference theory in which predictive processing operates similarly for production and perception, using a common set of predictions derived from a shared generative model (as suggested in Fig. 1B).

There are however some areas in which further work is needed to successfully extend active inference accounts to speech. One priority is to create and test computational models of active inference that can simulate speech motor adaptation and compensation responses to perturbations of speech sensory feedback. This would enable the development of more specific and testable hypotheses such as the necessity of proprioception for different aspects of speech motor control, and speech motor learning. In previously published simulations, Najnin and Banerjee (2017) demonstrate how a computational active inference framework can provide a superior account of the development of speech motor control. Such a computational model was found to demonstrate self-organisation of developmental stages, progressing from an initial babbling phase to the emergence of vowels and syllables. It further learned the timing of execution of motor commands automatically by estimating the transition time required to reach a proprioceptive target (in contrast to previous similar models in which timing was fixed; e.g., Moulin-Frier et al., 2014). Finally, such a model implemented multimodal integration of auditory and proprioceptive sensations, providing a clear pathway for auditory prediction error to result in changes to action through updating of proprioceptive predictions. Further development of this model would allow for simulation of the effects of perception of other voices on speech production in phenomena such as phonetic convergence, as well as interactions between convergence and speech motor adaptation/compensation (as shown by Bradshaw et al., 2024). These models therefore have great promise in providing an integrated account of speech motor control, from imitation from the environment during speech development through to multitalker interactions in the mature system.

Future active inference models of speech should also focus on applying the framework to the use of tactile inputs in speech motor control. This paper has predominantly focused on auditorily guided speech production as a useful starting point, given the prevailing view that the primary goals of speech lie in the auditory domain (Perrier, 2005). However, tactile feedback also plays a crucial role in articulation of speech sounds; for example, contact between articulatory surfaces can provide critical information on the timing of articulatory gestures, particularly during the production of consonants (Kent, 2024). Indeed, mechanical perturbations which alter speech somatosensory feedback (both tactile and proprioceptive) induce compensatory responses, even when such perturbations are designed so as to not affect auditory feedback (Honda et al., 2002; Nasir & Ostry, 2006, 2008; Smith et al., 2020; Tremblay et al., 2003, 2008). While active inference distinguishes between proprioceptive and tactile feedback (given proprioception’s privileged position in being able to engage motor reflexes to directly drive movement), the pathways and processes illustrated in this paper for auditory prediction error minimisation would be expected by active inference to apply equally to the minimisation of tactile prediction errors (and indeed other forms of relevant exteroceptive feedback). Active inference computational models should therefore be well equipped to simulate responses to mechanical perturbations that affect somatosensory feedback, though these are outside the scope of the current paper.

A further critical step will be to map active inference computational models for speech onto explicit neural architectures. Indeed, a key appeal of active inference for other motor domains lies in its potential to provide a neurally specified framework that is biologically plausible, and able to account for a range of neuroimaging and neuropsychological data (Friston, FitzGerald, et al., 2017a, 2017b; Friston, Parr, et al., 2017a, 2017b; Parr & Friston, 2018; Parr et al., 2025; Walsh et al., 2020). This neural specification has been demonstrated for a range of sensorimotor domains outside of speech, such as handwriting (Friston et al., 2011), oculomotor control (Perrinet et al., 2014), and visuomotor adaptation (Limanowski, 2022b). Developing similar, neural models for speech sensorimotor control would allow for the development of more specific hypotheses that can be tested with neural data, to evaluate the biological plausibility of active inference theories for speech. Example areas of investigation include neural implementation of the multimodal integration of auditory and proprioceptive sensations for speech or of hypothesised precision weighting mechanisms during adaptation and compensation to speech sensory feedback perturbations. Such computationally grounded neural models would also offer the opportunity to develop predictive coding accounts of disorders of speech motor control. For example, developmental stuttering has long been hypothesised to involve problems in predicting speech sensory feedback, in part based on observations of impaired speech motor adaptation and atypical evoked responses to auditory probes presented during periods of speech motor planning (Bradshaw et al., 2021; Max & Daliri, 2019; Max et al., 2004). As highlighted in Point 3 above, active inference would expect that such disruption to the prediction of self-generated speech auditory feedback during production should also be manifest during prediction of speech auditory input produced by other speakers during perception. Interestingly, recent evidence suggests that people who stutter do indeed show atypical neural markers of predictive mechanisms during speech perception as well as production (Gastaldon et al., 2023, 2024), in line with assumptions of predictive coding accounts. This framework may thus offer the potential for more holistic accounts of disorders affecting speech motor control, and their relation to other motor-control disorders such as Parkinson’s disease.

More research is also needed to investigate the role played by interactions between auditory and proprioceptive sensory feedback during speech production, and how these two sources of feedback might be integrated into multimodal predictions. Drawing on the active inference framework, research on visuomotor control of limb movement has proposed that visual feedback is afforded more sensory precision than proprioceptive feedback when forming a multimodal estimate of limb position (Limanowski, 2022a, 2022b). Hence, conflicts between visual and proprioceptive feedback (e.g., via external perturbations) are resolved via an updating of proprioceptive ‘beliefs’ (e.g., about hand position; Limanowski & Friston, 2020). This is conceptually similar to how the SFC/FACTS model accounts for responses to auditory feedback perturbations, in which auditory prediction errors are used to update the state estimate (i.e., the ‘belief’ as to the current positions of the articulators/constriction of the vocal tract).

By contrast, the active inference account of adaptation put forward in the current paper follows the framework for multimodal integration provided by Najnin and Banerjee (2017), in which auditory prediction errors are used to update the generative model’s ‘belief’ as to what auditory outcome is associated with the current proprioceptive state, itself directly signalled by proprioceptive feedback (rather than indirectly estimated). Experimental manipulations that modify the reliability and thus the precision of proprioceptive versus auditory feedback during speech (e.g., sensory degradation techniques such as topical oral anaesthesia or real-time spectral degradation as in Casserly 2015; Casserly & Marino, 2024; De Letter et al., 2020) could be employed to test for effects of sensory precision on speech motor adaptation, compensation and phonetic convergence. This would be a fruitful avenue for empirically testing active inference accounts in the domain of speech, given the importance of precision weighting in this proposal.

More widely, there is a need to reconsider the dominance of auditory prediction errors in current accounts of speech motor adaptation, and to explore alternative conceptualisations of this process in the multimodal domain; particularly considering the (relatively neglected) role of proprioception. Interestingly, a similar argument has recently been made for understanding visuomotor adaptation (Tsay et al., 2022). The idea of differences in precision weighting of proprioceptive versus auditory feedback may also be able to explain individual variability in speech motor adaptation to auditory versus somatosensory/proprioceptive (jaw position) perturbations, which are negatively correlated within individuals (Lametti et al., 2012). Interestingly, these sensory preferences have been modelled within the latest version of the GEPPETO model (Patri et al., 2019). One implementation involved changes to the ‘precision’ of sensory target distributions; however, the authors characterised this as a stable trait of a speaker that wouldn’t be modulated across time. This contrasts with the moment-by-moment contextual flexibility assumed to characterise precision weighting within active inference accounts of limb motor control (Chancel & Ehrsson, 2023; Limanowski, 2022b). The application of active inference concepts from other domains thus offers the potential for new perspectives on key phenomena in speech motor control.

Overall, by explicitly comparing current approaches in speech motor control with the new frameworks offered by predictive coding and active inference, this review has sought to facilitate improved interdisciplinary thinking on the mechanisms underlying perception and action for speech. We hope that our preliminary descriptions of how an active inference theory of speech motor control might function can pave the way for further theorising, computational specification and empirical testing of these emerging hypotheses.

## Data availability (data transparency)

Not applicable (no data).

## Code availability (software application or custom code)

Not applicable (no data analysis).

## Appendix 1: Neural implementations of precision weighting for sensorimotor control

Active inference proposes that the precision of different distributions within generative models is associated with different neuromodulatory systems in the brain. For example, the cholinergic system is proposed to be associated with signalling the precision of sensory evidence; the serotonergic system with the precision of prior predictions; the noradrenergic system with the precision of transitions over time; and, particularly relevant for the topic of this paper, the dopaminergic system with the precision of different action plans (termed ‘policies’ in active inference) (for a review of evidence for these proposals, see Parr et al., 2022). For the latter example, dopaminergic signals from midbrain structures (the substantia nigra and ventral tegmental area) modulate the gain of signals in medium spiny neurons in the striatum; this modulation serves to balance activity in the direct and indirect pathways through the basal ganglia, in order to set a prior belief about the precision of different action plans (i.e. confidence in the utility of different actions for minimising prediction error), that are encoded in basal ganglia output nuclei (Parr et al., 2025). These in turn modulate the dendrites in superficial cortical layers via the thalamus (Shipp, 2007). Descending corticospinal projections then serve to attenuate the precision or gain of reflex arcs at the periphery (Brown et al., 2013), thus weighting the effect of proprioceptive prediction errors on motor output.

This neural architecture thus supports the selection of alternative motor plans, and sets the precision of the resulting proprioceptive predictions sent from motor cortex. Accordingly, disturbances of dopaminergic signalling lead to disturbances in this precision weighting process; for example, the akinesia seen in Parkinson’s disease patients is framed as a loss of confidence in policies, whereas hyperkinetic movements in Huntingdon’s disease are attributed to overconfidence in the selection of unintended action plans (Parr et al., 2025).

Neural oscillations have also been linked to precision weighting; for example, gamma-band activity has been associated with precision-weighted prediction errors, while beta-band activity has been linked to the precision of predictions (Richter et al., 2017; Sedley et al., 2016; Walsh et al., 2020). In the realm of motor control, Palmer et al. (2019) presented electrophysiological evidence for encoding of precision in beta activity during performance of a visuomotor adaptation task. Using computational modelling of adaptation behaviour, they found that beta power prior to a movement was modulated by model estimates of the precision of predictions, whereas beta power after a movement correlated with the precision of sensory prediction errors. Beta power has further been shown to be modulated by dopamine (Iskhakova et al., 2021; Jenkinson & Brown, 2011), providing a potential mechanism by which precision can be encoded through interactions between neuromodulatory and electrophysiological mechanisms.
